# A Secret Worth Practicing

**Published:** 1861-12

**Authors:** 


**A SECRET WORTH PRACTICING.**—When you've got any thing to do, do it. What an inconceivable number of unpleasant mental conditions and endurances might be avoided daily, if this homely rule were put in practice! When a thing has to be done, be decided and courageous enough to do it on the spot, and have it off your mind. Procrastination is cowardice—the pulling of a tooth for example; as also the taking a whole dollar out of pocket on the instant, and sending it for the JOURNAL for next year. It has to be done, reader; and you are determined to do it. What is the use, then, of putting it off, it may be for a month, or more, and then sending it along with an excuse, that you "intended" etc. etc. We know very well that you can't well do without it, hence we feel a little independence in the premises. And while you are at it, send an extra dollar for your minister, as a New-Year's gift.

